# Risk of eating disorders in relation to stress in a sample of Egyptian students in Ain-Shams university

**DOI:** 10.1186/s40337-026-01550-9

**Published:** 2026-03-08

**Authors:** Tarek Okasha, Rehab Mohamed Naguib, Ahmed Zaki, Nahla Dessouki

**Affiliations:** Okasha institute of Psychiatry, Faculty of Medicine, Ain Shams University, Cairo, Egypt

**Keywords:** Eating disorders, Medical students, Perceived stress, EAT-26, Risk factors

## Abstract

**Background:**

Eating disorders (EDs) are serious psychiatric conditions with increasing prevalence among university students, often linked to high stress levels and unhealthy coping mechanisms. Medical students are particularly susceptible due to their demanding academic environment. This study aimed to assess the prevalence of EDs risk among medical and non-medical undergraduate students and examine its association with perceived stress and related factors.

**Methods:**

A comparative cross-sectional study was conducted on 319 undergraduate students aged 17–24 years from Ain Shams University, including 173 medical and 146 non-medical students. Participants completed validated Arabic versions of the Eating Attitudes Test (EAT-26), Eating Disorder Examination Questionnaire (EDE-Q), and Perceived Stress Scale (PSS). Students were classified into high- and low-risk groups for EDs based on EAT-26 scores.

**Results:**

EDs risk was significantly higher in medical students (35.8%) compared to non-medical students (24.7%, *P* = 0.031). Medical students also showed higher perceived stress scores (19.87 ± 7.46 vs. 17.03 ± 7.32, *P* < 0.001) and more frequent severe stress (24.3% vs. 11%, *P* = 0.006). EDE-Q scores for restraint, shape concern, weight concern, and global score were significantly higher in medical students. High-risk students had elevated BMI (25.32 ± 4.06 vs. 23.70 ± 3.36, *P* = 0.001) and higher smoking prevalence (39.8% vs. 9.5%, *P* < 0.001). Severe stress (OR = 2.091) and smoking (OR = 9.208) were independent predictors of EDs risk.

**Conclusions:**

Medical students are at elevated risk of EDs, largely driven by high perceived stress and unhealthy lifestyle behaviors.

## Abstract (plain language summary)

Eating disorders are severe mental health conditions that disrupt normal attitudes and behaviors related to food, weight, and body perception. They often begin in young people during university, when stress is high. This investigation assessed if elevated stress levels contribute to a higher risk of eating disorders among Egyptian undergraduates. We compared medical students, who usually face intense academic pressure, with students from non-medical fields. We found that medical students had higher levels of stress and were more likely to show signs of eating disorder risk. Students who smoked or reported very high stress were at greatest risk.

These results focus on the need for better mental health support and healthier coping strategies for students, especially those in demanding academic programs. Early help may prevent serious eating problems later on.

## Introduction

Eating disorders (EDs), including bulimia nervosa, anorexia nervosa, and binge-eating disorder, are complex psychiatric conditions characterized by disturbances in eating behavior, body image, and weight perception. These disorders typically emerge during adolescence and early adulthood, coinciding with the period when many individuals begin university [1]. Globally, the EDs burden has increased in recent years, with university students recognized as a particularly vulnerable group due to developmental, psychological, and social pressures. EDs are associated with significant morbidity, including nutritional deficiencies, psychological distress, impaired academic performance, and in severe cases, increased mortality risk [2–4].

Among the key psychological contributors to disordered eating is perceived stress. Stress, defined as a state of mental or emotional strain resulting from demanding circumstances, has been strongly linked to emotional dysregulation, negative coping mechanisms, and altered appetite control [5]. The psychosomatic theory of eating behavior suggests that individuals experiencing emotional stress may turn to food not out of physiological hunger, but as a coping strategy to regulate affect. This maladaptive response can manifest as restrictive eating, bingeing, or purging, contributing to the development and persistence of EDs. In the university setting, stressors such as academic workload, social integration, financial concerns, and uncertainty about future careers may increase the risk of EDs, particularly when healthy coping mechanisms are lacking [6].

Medical students, in particular, experience disproportionately high levels of stress compared to their non-medical peers. The intensity of medical education, characterized by long study hours, frequent evaluations, exposure to human suffering, and high expectations, places these students at an elevated risk for burnout, anxiety, depression, and disordered eating [7, 8]. Several studies have reported a higher prevalence of EDs in medical students compared to the general population, raising concerns about the mental well-being of future healthcare providers and the need for early identification and intervention within this demographic [9, 10].

A substantial body of literature has established a link between perceived stress and maladaptive eating behaviors in student populations and there is a notable lack of evidence from Middle Eastern/North African university settings, where cultural norms around stress, body image and mental health stigma may distinctly shape this association. This represents a significant contextual gap. In light of these considerations, this study aimed to estimate the prevalence of EDs risk among medical and non-medical students at Ain Shams University (in the same institution), using multiple validated tools to assess the relationship between perceived stress and EDs risk, and to compare associated risk factors across both groups. Understanding these dynamics is crucial to develop culturally competent targeted prevention strategies and enhance student mental health support systems.

## Subjects and methods

### Study design and setting

This comparative cross-sectional study was carried out at Ain Shams University, Cairo, Egypt, including students from the Faculty of Medicine and the Faculty of Arts. Medicine and Arts faculties were chosen because they typically represent contrasting environments which was intended to maximize the potential for observing differences in lifestyle-related outcomes. Sampling from these two, well-defined faculties ensured proper recruitment and adequate sampling within the study timeline. Data collection was carried out over a 10-month period, from April 2024 to February 2025. The study was reviewed and approved by the Research Ethics Committee, Ain Shams University Faculty of Medicine, and written informed consent was obtained from all participants included.

### Study population and eligibility criteria

The study included undergraduate students from both faculties who met the following inclusion criteria: aged between 17 and 24 years, of both sexes, and willing to participate through signed informed consent. Students were excluded if they had any known general medical or neurological condition, were taking medications that affect appetite, or screened positive for psychiatric illness using the General Health Questionnaire (GHQ-28).

### Grouping

Participants were classified into two groups: Group A (*n* = 173): Medical students from the Faculty of Medicine, and Group B (*n* = 146): Non-medical students from the Faculty of Arts.

#### Study procedure

**Fig. 1:**
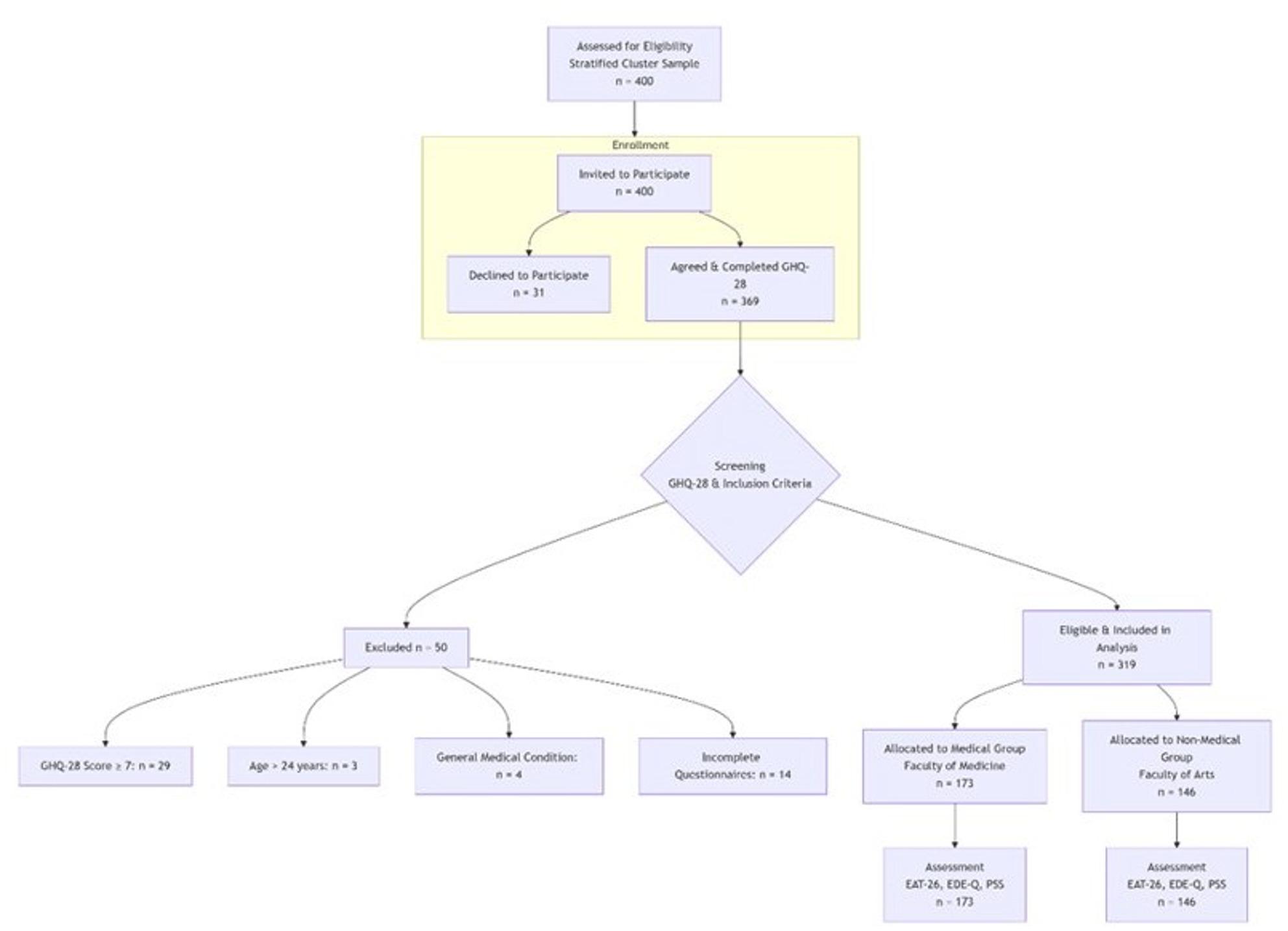
Flow diagram of participant selection and allocation

This study employed a stratified cluster sampling technique to recruit undergraduate students from two distinct faculties at Ain Shams University: The Faculty of Medicine and the Faculty of Arts. The primary aim was to compare eating disorder risk, associated attitudes, and perceived stress levels between medical and non-medical student populations. A total of 400 students were formally invited to participate. Of these, 369 students provided consent and completed the initial screening instrument. All participants first completed a predesigned information sheet and the 28-item General Health Questionnaire (GHQ-28). To establish a sample without significant general psychological distress—a potential confounding factor—participants scoring ≥ 7 on the GHQ-28 were excluded from further assessment (*n* = 29). Additional exclusion criteria were applied: being over 24 years of age (*n* = 3), having a known general medical condition (*n* = 4), and failing to complete the subsequent required questionnaires (*n* = 14). Consequently, the final analytical sample comprised 319 students who met all inclusion/exclusion criteria. These participants were categorized into a Medical Student group (*n* = 173, from the Faculty of Medicine) and a Non-Medical Student group (*n* = 146, from the Faculty of Arts). The participant flow is detailed in Fig. 1.

### Sampling method and sample size calculation

A stratified cluster sampling technique was employed. Students were first stratified by academic year (first to fifth year for medical students and first to fourth year for arts students). From each year, one class section was randomly selected, and all students present during data collection were invited to participate. To compensate for potential incomplete responses, the initial sample size was increased.

Using PASS 15 software (version 15.0.10), and based on prior data by Ngan et al. [11] suggesting an 11% prevalence of EDs risk among medical students, the required minimum sample size was calculated. With a 90% confidence level, ± 5% margin of error, and accounting for a 10% dropout rate, a sample of at least 140 students per group was deemed sufficient.

### Study tools and measures

Participants completed a structured data collection sheet and a battery of validated Arabic-translated questionnaires as follows:

#### Sociodemographic data sheet

Captured age, gender, BMI, academic year, smoking status, residence, marital status, parental education and occupation, and exercise habits.

#### General health questionnaire (GHQ-28)

Used to screen for psychiatric illness and exclude ineligible participants. The Arabic version validated by Okasha [12] was used, with a cut-off score of ≥ 7 indicating likely psychological morbidity [13].

#### Eating attitudes test (EAT-26)

A 26-item screening tool for disordered eating risk, covering dieting, bulimia/food preoccupation, and oral control domains. The Arabic version validated by Haddad et al. [14] was utilized. The Arabic EAT-26 has demonstrated good internal consistency (Cronbach’s α = 0·895).

#### Eating disorder examination questionnaire (EDE-Q)

A 28-item scale assessing core features of EDs psychopathology across restraint, shape concern, eating concern, and weight concern subscales. The validated Arabic version by Aldubayan et al. [15] was used. The Arabic EDE-Q has shown excellent reliability (The internal consistency of the global and the three subscales were high, with Cronbach’s α ranging from 0.762 to 0.900).

#### Perceived stress scale (PSS)

A 10-item tool to measure perceived stress over the past month based on Cohen et al. [16]. The Arabic version validated by Chayaa et al. [17] was applied. It has established good reliability (Internal consistency of the PSS-10 and its positive and negative subscales was acceptable (coefficient α = 0.67, 0.79, and 0.86, respectively). Since the PSS-10 lacks validated clinical cutoffs, perceived stress severity was categorized using sample-based percentiles:

- Mild stress (≤ 25th percentile; scores 0–12),
- Moderate stress (26th–75th percentile; 13–28),
- Severe stress (> 75th percentile; 29–40).

This approach aligns with regional studies [17] that used distribution-based categorization when standardized thresholds were unavailable.

### Study procedure

A total of 400 students were approached; 369 agreed to participate. Following administration of the GHQ-28, 29 students were excluded due to scoring ≥ 7. Additional exclusions included 3 students over the age of 24, 4 with general medical conditions, and 14 with incomplete data, resulting in a final sample of 319 participants (173 medical and 146 non-medical students).

Eligible participants (GHQ score < 7) proceeded to complete the EAT-26, EDE-Q, and PSS questionnaires. The EAT-26 was used to screen for EDs risk, while the EDE-Q provided detailed assessment of eating-related behaviors and attitudes. The PSS measured perceived stress levels across the cohort.

Statistical methods.

All data handling and analyses were carried out using SPSS software, version 23.0 (IBM Corp., Chicago, IL, USA). The distribution of variables was examined with the Kolmogorov–Smirnov and Shapiro–Wilk tests. Continuous variables conforming to a normal distribution were expressed as mean ± standard deviation together with their range, whereas skewed data were summarized as median values with interquartile ranges. Categorical variables were described using absolute numbers and percentages. Between-group differences in normally distributed continuous variables were assessed using the independent samples t-test, while categorical comparisons were performed with the Chi-square test. Associations among ordinal variables were determined using Spearman’s rank correlation coefficient. A two-sided P-value < 0.05 was taken to indicate statistical significance, with results reported at the 95% confidence level. Variables with *p* < 0.05 in univariate analyses were entered into a multivariate model to identify independent predictors of eating risk among the studied group.

## Results

The studied participants had a mean age of 19.85 ± 1.53 years, with a nearly equal gender distribution (51.1% males and 48.9% females). Medical students represented 54.2% of the sample, while non-medical students accounted for 45.8%. The average weight and height were 69.28 ± 10.91 kg and 169.30 ± 5.45 cm, respectively, with a mean BMI of 24.20 ± 3.66 kg/m². Smokers constituted 30.1% of the cohort. Most students were in their first or second academic year (25.4% and 26%, respectively). The majority were single (90.9%) and lived in urban areas (68.3%). Regarding parental education, 76.5% of fathers and 66.5% of mothers had university or higher education. Professional occupations were the most common among fathers (45.1%) and mothers (27.6%), while 56.4% of mothers were nonworking. Only 29.5% of participants reported practicing exercise. Table 1.

**Table 1:** General characteristics of the studied participants (*n* = 319)

General characteristics	
Faculty	n (%)
Medical	173 (54.2)
Non-medical	146 (45.8)
Age (years)	Mean ± SD
	19.85 ± 1.53
Gender	n (%)
Male	163 (51.1)
Female	156 (48.9)
	Mean ± SD
Weight (kg)	69.28 ± 10.91
Height (cm)	169.30 ± 5.45
BMI (kg/m²)	24.20 ± 3.66
	n (%)
Smoking	96 (30.1)
Academic year	
First	81 (25.4)
Second	83 (26)
Third	58 (18.2)
Fourth	57 (17.9)
Fifth	40 (12.5)
Marital status	
Single	290 (90.9)
Married	20 (6.3)
Residence	
Urban	218 (68.3)
Rural	101 (31.7)
Paternal education	
Secondary or lower	75 (23.5)
University and higher	244 (76.5)
Paternal occupation	
Nonworking	23 (7.2)
Manual	51 (15.9)
Clerical	101 (31.7)
Professional	144 (45.1)
Maternal education	
Secondary or lower	107 (33.5)
University and higher	212 (66.5)
Maternal occupation	
Nonworking	180 (56.4)
Skilled / Manual / Clerical	51 (16)
Professional	88 (27.6)
Practice of exercise	94 (29.5)

*n* number,* SD* Standard deviation,* BMI* Body Mass Index,* kg* kilogram,* cm* centimeter,* m²* square meter,* %* percentage

Medical students exhibited markedly higher perceived stress scores compared to non-medical students (19.87 ± 7.46 vs. 17.03 ± 7.32, *P* < 0.001). Severe stress was more prevalent among medical students (24.3%) than among non-medical students (11%), while mild stress was more common in the non-medical group (26.7% vs. 13.3%), indicating a statistically significant difference in stress severity distribution (*P* = 0.006). Additionally, a higher proportion of medical students were classified as high-risk for EDs based on the EAT-26 score (35.8% vs. 24.7%, *P* = 0.031). Regarding disordered eating behaviors assessed by the EDE-Q, medical students had significantly higher mean scores in restraint (1.33 ± 0.77 vs. 1.13 ± 0.67, *P* = 0.014), shape concern (2.73 ± 1.10 vs. 2.44 ± 0.93, *P* = 0.012), weight concern (1.79 ± 1.09 vs. 1.48 ± 0.88, *P* = 0.005), and global score (1.68 ± 0.86 vs. 1.46 ± 0.74, *P* = 0.015). There was no significant difference between the two groups regarding eating concern (*P* = 0.137). Table 2.

**Table 2:** Distribution of perceived stress and eds risk according to EAT-26 and eds behavior and attitude according to EDE-Q questionnaire among medical and non-medical students

PSS	Medical (*n* = 173)	Non-medical (*n* = 146)	*P*-value	t/ χ^2^
	Mean ± SD			
	19.87 ± 7.46	17.03 ± 7.32	< 0.001*	3.417
EDs risk according to EAT-26				
Stress severity	n (%)			
Mild	23 (13.3)	39 (26.7)	< 0.001*	15.059
Moderate	108 (62.4)	91 (62.3)		
Severe	42 (24.3)	16 (11)		
Low-risk (≤ score 20)	111 (64.2)	110 (75.3)	0.031*	4.651
High-risk (> score 20)	62 (35.8)	36 (24.7)		
EDs behavior and attitude according to EDE-Q	Mean ± SD			
Restraint	1.33 ± 0.77	1.13 ± 0.67	0.014*	2.480
Eating concern	0.88 ± 0.65	0.78 ± 0.64	0.169	1.379
Shape concern	2.73 ± 1.10	2.44 ± 0.93	0.012*	2.516
Weight concern	1.79 ± 1.09	1.48 ± 0.88	0.005*	2.810
Global score	1.68 ± 0.86	1.46 ± 0.74	0.015*	2.456

*PSS* Perceived Stress Scale,* SD* Standard Deviation,* n* number,* EAT-26* Eating Attitudes Test-26,* EDE-Q* Eating Disorder Examination Questionnaire, * Significant P-value.* t* t value of independent t-test. χ^2^: χ^2^ value of Chi square test

Individuals were categorized into two groups based on their EDs risk according to the EAT-26 questionnaire: a low-risk group (*n* = 221) and a high-risk group (*n* = 98).

Compared to the low-risk group, high-risk participants had significantly higher mean body weight (72.98 ± 11.55 vs. 67.63 ± 10.21 kg, *P* < 0.001) and BMI (25.32 ± 4.06 vs. 23.70 ± 3.36 kg/m², *P* = 0.001). Smoking was also significantly more prevalent among high-risk participants (39.8% vs. 9.5%, *P* < 0.001). Academic year distribution differed significantly between the groups, with a greater proportion of high-risk students in the fourth year (29.6% vs. 12.7%) and fewer in the first and third years (*P* = 0.002). Moreover, regular physical exercise was reported significantly less among high-risk students compared to those at low risk (21.4% vs. 33%, *P* = 0.036). No notable variations were observed between the two groups regarding age (*P* = 0.527), gender (*P* = 0.14), height (*P* = 0.346), marital status (*P* = 0.153), socioeconomic status (*P* = 0.225), residence (*P* = 0.19), paternal education (*P* = 0.575), paternal occupation (*P* = 0.243), maternal education (*P* = 0.63), or maternal occupation (*P* = 0.13) Table 3.

**Table 3:** Relation between eds risk and patient characteristics between high-and low-risk groups

Age (years)		Low-risk (*n* = 221)	High-risk (*n* = 98)	*P*-value	t/ χ^2^
	Mean ± SD	19.81 ± 1.57	19.93 ± 1.44	0.527	0.646
Gender					
Male	n (%)	119 (53.8)	44 (44.9)	0.140	2.176
Female		102 (46.2)	54 (55.1)		
Weight (kg)	Mean ± SD	67.63 ± 10.21	72.98 ± 11.55	< 0.001*	4.144
Height (cm)		169.00 ± 8.51	170.00 ± 8.42	0.346	0.971
BMI (kg/m²)	Mean ± SD	23.70 ± 3.36	25.32 ± 4.06	< 0.001*	3.720
n (%)					
Smoking		21 (9.5)	39 (39.8)	< 0.001*	40.800
Academic year	n (%)				
First		62 (28.1)	19 (19.4)	0.002*	
Second		55 (24.9)	28 (28.6)		
Third		47 (21.3)	11 (11.2)		17.204
Fourth		28 (12.7)	29 (29.6)		
Fifth		29 (13.1)	11 (11.2)		
Marital status	n (%)				
Single		210 (95)	89 (90.8)	0.153	2.044
Married		11 (5)	9 (9.2)		
Socioeconomic state	n (%)				
Low		60 (27.1)	36 (36.7)	0.225	
Moderate		108 (48.9)	41 (41.8)		2.982
High		53 (24)	21 (21.4)		
Residence					
Urban		146 (66.1)	72 (73.5)	0.19	1.721
Rural		75 (33.9)	26 (26.5)		
Paternal education					
Secondary or lower		50 (22.6)	25 (25.5)	0.575	0.314
University and higher		171 (77.4)	73 (74.5)		
Paternal occupation					
Nonworking		17 (7.7)	6 (6.1)	0.243	4.174
Manual		41 (18.6)	10 (10.2)		
Clerical		66 (29.9)	35 (35.7)		
Professional		97 (43.9)	47 (48)		
Maternal education					
Secondary or lower		76 (34.4)	31 (31.6)	0.63	0.231
University and higher		145 (65.6)	67 (68.4)		
Maternal occupation					
Nonworking		125 (56.6)	55 (56.1)	0.13	5.650
Manual		5 (2.3)	4 (4.1)		
Clerical		24 (10.9)	18 (18.4)		
Professional		67 (30.3)	21 (21.4)		
Practice of exercise		73 (33)	21 (21.4)	0.036*	4.398

*n* number,* SD* Standard Deviation,* kg* kilogram, cm: centimeter,* BMI* Body Mass Index,* m²* square meter,* %* percentage, * Significant P-value.* t* t value of independent t-test. χ^2^: χ^2^ value of Chi square test

Students at high risk of EDs, as determined by the EAT-26, demonstrated significantly higher perceived stress scores compared to those at low risk (25.92 ± 6.07 vs. 15.32 ± 5.55, *P* < 0.001). Moreover, stress severity showed a significant association with EDs risk. Severe stress was markedly more prevalent among high-risk students (49% vs. 4.5%), while mild stress was reported exclusively in the low-risk group (28.1% vs. 0%), with a significant difference in distribution (*P* < 0.001). Table 4.

**Table 4:** EDs risk using EAT-26 in relation to perceived stress using PSS between high-and low-risk groups

		Low-risk (*n* = 221)	High-risk (*n* = 98)	*P*-value	t/ χ^2^
PSS	Mean ± SD	15.32 ± 5.55	25.92 ± 6.07	< 0.001*	15.285
Stress severity	n (%)				
Mild		62 (28.1%)	0 (0%)	< 0.001*	
Moderate	n (%)	149 (67.4%)	50 (51%)		104.215
Severe	n (%)	10 (4.5%)	48 (49%)		

*PSS* Perceived Stress Scale, *SD* Standard Deviation, *n* number, *%* percentage, *: Significant P-value. *t* t value of independent t-test. χ^2^: χ^2^ value of Chi square test

Perceived stress scores revealed significant positive correlations with all EDE-Q domains, including restraint (*r* = 0.744, *P* < 0.001), eating concern (*r* = 0.713, *P* < 0.001), shape concern (*r* = 0.741, *P* < 0.001), weight concern (*r* = 0.754, *P* < 0.001), and global score (*r* = 0.781, *P* < 0.001). Body weight also showed significant positive correlations with restraint (*r* = 0.240, *P* < 0.001), eating concern (*r* = 0.259, *P* < 0.001), shape concern (*r* = 0.232, *P* < 0.001), weight concern (*r* = 0.225, *P* < 0.001), and global score (*r* = 0.250, *P* < 0.001). Similarly, BMI was significantly correlated with restraint (*r* = 0.232, *P* < 0.001), eating concern (*r* = 0.268, *P* < 0.001), shape concern (*r* = 0.209, *P* < 0.001), weight concern (*r* = 0.228, *P* < 0.001), and global score (*r* = 0.243, *P* < 0.001). In contrast, no significant correlations were observed between EDE-Q domains and age (*P* = 0.529, 0.941, 0.366, 0.475, and 0.524, respectively) or height (*P* = 0.618, 0.771, 0.309, 0.815, and 0.570, respectively) Table 5.

**Table 5:** Correlation between EDE-Q and demographic data and stress

Stress score	Restraint	Eating concern	Shape concern	Weight concern	Global score
r	0.744	0.713	0.741	0.754	0.781
P-value	< 0.001*	< 0.001*	< 0.001*	< 0.001*	< 0.001*
Age (year)					
r	0.035	-0.004	0.051	0.04	0.036
P-value	0.529	0.941	0.366	0.475	0.524
Weight (kg)					
r	0.24	0.259	0.232	0.225	0.25
P-value	< 0.001*	< 0.001*	< 0.001*	< 0.001*	< 0.001*
Height (cm)					
r	0.028	0.016	0.057	0.013	0.032
P-value	0.618	0.771	0.309	0.815	0.57
BMI					
r	0.232	0.268	0.209	0.228	0.243
P-value	< 0.001*	< 0.001*	< 0.001*	< 0.001*	< 0.001*

*EDE-Q* Eating Disorder Examination Questionnaire, *r* correlation coefficient, *BMI* Body Mass Index, *kg* kilogram, *cm* centimeter, * Significant P-value

Multivariate logistic regression analysis revealed that severe stress was a significant predictor of EDs risk, with students experiencing severe stress having approximately double the odds of being at high risk (OR = 2.091, 95% CI 1.543–2.835, *P* < 0.001). Smoking was also a strong independent predictor, with smokers being over nine times more likely to be at high risk for EDs compared to non-smokers (OR = 9.208, 95% CI 4.038–20.997, *P* < 0.001) Table 6.

**Table 6:** Multivariate logistic regression analysis for predictors of eating risk among the studied group

Severe stress	OR (95% CI)	*P*-value
	2.091 (1.543–2.835)	< 0.001*
Smoking	9.208 (4.038–20.997)	< 0.001*
Exercise	0.507 (0.215–1.194)	0.12
Fourth academic year	1.498 (0.639–3.511)	0.352

*OR* Odds Ratio, *CI* Confidence Interval, * Significant P-value

## Discussion

EDs are serious psychiatric conditions marked by maladaptive eating behaviors, distorted body image, and significant psychological and physical morbidity. These disorders, most commonly beginning in adolescence and early adulthood, are increasingly prevalent among university students and are frequently linked to stress, sociocultural pressure, and poor coping mechanisms. Understanding the relationship between stress and EDs in student populations, particularly those exposed to high academic loads such as medical students, is essential for early identification and effective intervention strategies [18, 19].

In the current study involving 319 undergraduate students aged 17–24 years, we found that 35.8% of medical students and 24.7% of non-medical students were at high risk of EDs according to the EAT-26. Medical students also demonstrated significantly higher stress scores compared to non-medical students, and higher frequencies of severe stress. Additionally, mean EDE-Q scores (shape concern, restraint, weight concern, and global score) were significantly higher in medical students. High-risk EDs individuals had higher BMI, body weight, perceived stress, and were more likely to be smokers and fourth-year students. Multivariate analysis identified severe stress and smoking as significant independent predictors of EDs risk.

Regarding the prevalence of EDs risk, our findings showed a higher prevalence of EDs risk among medical students (35.8%) compared to non-medical students (24.7%), supporting prior research. Iyer and Shriraam [20] reported a 22.7% prevalence among medical students, while Ali and Shehata (2020) found 33% of medical students were at risk. Similarly, Bizri et al. [21] found 17% of medical students at the American University of Beirut were at high risk based on EAT-26. These rates align with our findings and highlight the consistent vulnerability of medical students to disordered eating. In contrast, Chan et al. [22] and Tavolacci et al. [23] reported lower rates (13.9% and 20.5%, respectively), possibly due to cultural, environmental, or academic context variations.

Regarding stress levels, our data demonstrated that medical students experienced significantly higher perceived stress levels than non-medical peers, with severe stress reported in 24.3% versus 11%. This is consistent with Aamir [24] and Husnain [25], who both found significantly elevated stress in medical students compared to non-medical peers. Ngan et al. [11] also reported that over 40% of medical students experienced high stress. These findings underscore the heightened psychological burden among medical students compared to age-matched peers. That arise from the intense academic pressure, emotional strain of clinical practice on the students, and subsequent lack of work-life balance characteristic of medical training. Another pervasive factor is the stigma around mental health within medical culture which often delays help-seeking. In addition to the financial stressors and repetitive circadian rhythm disruption and sleep deprivation that further compound the stress risk.

Regarding the relationship between stress and EDs risk, there was a clear association between higher perceived stress and increased EDs risk, with high-risk students scoring significantly higher on the PSS. This supports findings by Iyer and Shriraam [20], Girlani et al. [26], and Jahrami et al. [27], all of whom reported significant associations between stress and disordered eating. In contrast, Swamy [28] found no such correlation among college faculty members, possibly due to differences in life stage and stress sources. Ngan et al. [11] also found no significant association, potentially due to variations in academic stressors or coping mechanisms.

Regarding EDE-Q subscales and eating attitudes, medical students showed significantly higher EDE-Q scores in shape concern, restraint, weight concern, and global scores compared to non-medical students, suggesting greater body image dissatisfaction and maladaptive eating behaviors. These findings are consistent with Chaudhari et al. [29] and Thangaraju et al. [30], who reported high EDE-Q scores among medical students. Iyer and Shriraam [20] also reported that increased shape concern was substantially associated with higher EAT-26 scores.

Regarding BMI and weight, we found that students in the high-risk group had notably higher body weight and BMI. This aligns with studies by Kabakuş Aykut and Bilici [31], Chaudhari et al. [29], and Ali and Shehata [32], all of which reported that higher BMI was positively associated with EDs risk. These findings reinforce the bidirectional link between weight-related concerns and disordered eating behaviors.

Regarding physical activity, lack of exercise was significantly associated with increased EDs risk in our sample. Supporting this, Iyer and Shriraam [20] noted that students who did not engage in regular exercise had higher EAT-26 scores. Zhang et al. [33] and Eck et al. [34] have previously emphasized the protective role of physical activity in improving body image and self-esteem, factors closely linked to EDs development.

Regarding academic year, a significant association was found between fourth academic year and high EDs risk. This may be attributed to increased academic demands and transitional stress common in mid-program stages. This finding is indirectly supported by Husnain [20], who noted rising stress across academic years. Such stress may precipitate unhealthy coping behaviors including disordered eating.

Regarding smoking, smoking was strongly associated with EDs risk and identified as an independent predictor (OR = 9.208). This agrees with Tavolacci et al. [23], who found higher smoking prevalence in students with positive SCOFF results. Similarly, Solmi et al. [35] concluded through meta-analysis that individuals with EDs are more likely to be lifetime smokers. Nicotine’s appetite-suppressant effect may explain its use as a maladaptive weight control strategy [36]. Smoking is strongly linked to both impulsive and compulsive traits, which are central to the behavioral and emotional dysregulation associated with eating disorders. Engagement of older students in more cigarette smoking could be due to increased workload stress or to false perceived associations of smoking with maturity and social status, together with lack of proper training in smoking cessation programs to medical students. Which necessitates development and implementation of medical education curriculum guidelines that focus on tobacco awareness and cessation among students in order to reduce the rising smoking rates among future physicians.

Regarding stress and EDE-Q scores correlation, we observed strong positive correlations between stress scores and all EDE-Q subscales, indicating that higher stress levels were linked with more severe disordered eating behaviors. This is supported by Anderson [37] and Barayan et al. [38], who reported similar correlations in university populations. Klatzkin et al. [39] demonstrated that perceived stress positively correlates with binge eating frequency, further supporting our results. The strong positive correlations observed between perceived stress and all EDE-Q subscales in our study provide support for a psychosomatic pathway and allow for more potential mechanisms. The link with restraint suggests that for a subset of students, elevated stress may trigger maladaptive coping strategies as strict dietary control, which creates a sense of order and self-efficacy facing unpredictable academic demands. As for the positive correlations with eating, weight, and shape concerns, it supports the notion that stress may not only influence behavior but also amplifies profoundly the cognitive preoccupation and affective distress related to body image. Which aligns with the theory that stress can exacerbate underlying body dissatisfaction and dysmorphic thinking. In the context of our sample, the unique pressures faced by the undergraduate students may make them particularly vulnerable, as their physical appearance or self-presentation can feel subject to constant evaluation. Therefore, our data suggest that stress may actively fuel both the cognitive distortions and the behavioral attempts rather than merely co-occurrence with the disordered eating, highlighting the importance of stress-management interventions that also address the perception of body image.

Several limitations should be acknowledged. To begin with, the cross-sectional nature of the study restricts any conclusions about causal relationships between stress and the risk of eating disorders. Additionally, the sample being selected exclusively from the faculties of Medicine and Arts allowed for a focused comparison of two contrasting academic samples, however, it limits the generalizability of the findings to students in other faculties who may have different stress and lifestyle profiles. Also, the recruitment of participants from only one university reduces the extent to which the results can be generalized to wider student populations. Future research should aim for a more diverse range of faculties and universities to enhance the representativeness of the results. Furthermore, reliance on self-administered questionnaires introduces the possibility of recall errors and social desirability bias, which could influence response accuracy. Finally, unmeasured confounding variables such as dietary habits, media exposure, and underlying psychiatric conditions may have influenced the outcomes.

## Conclusions

Medical students are at elevated risk of EDs, largely driven by high perceived stress and unhealthy lifestyle behaviors. Early screening and targeted mental health support are essential.

## Data Availability

Available upon reasonable request.

## Abbreviations

BMI: Body Mass Index
CI: Confidence Interval
cm: Centimeter
EDs: Eating disorders
EAT-26: Eating Attitudes Test-26
EDE-Q: Eating Disorder Examination Questionnaire
GHQ-28: General Health Questionnaire-28
kg: Kilogram
m²: Square meter
n: Number
OR: Odds Ratio
PSS: Perceived Stress Scale
r: Correlation coefficient
SD: Standard Deviation

## References

[1] Camacho-Barcia L, Giel KE, Jiménez-Murcia S, Álvarez Pitti J, Micali N, Lucas I, et al. Eating disorders and obesity: bridging clinical, neurobiological, and therapeutic perspectives. Trends Mol Med. 2024;30:361–79. 10.1016/j.molmed.2024.02.007. 38485648 10.1016/j.molmed.2024.02.007

[2] Mushtaq T, Ashraf S, Hameed H, Irfan A, Shahid M, Kanwal R, et al. Prevalence of eating disorders and their association with social media addiction among youths. Nutrients. 2023;15. 10.3390/nu15214687. 10.3390/nu15214687 PMC10647586 37960340

[3] Anh T, Truc L, Linh N, Phuong N, Le N, Phuong N, et al. Prevalence of eating disorders and their association with depression, anxiety, and stress among high school students in suburban areas in Vi Etnam. J Adv Biotechnol Exp Ther. 2025;8:242. 10.5455/jabet.2025.20.

[4] Almahmeed MB, Almutawa MA, Naguib YM. The prevalence of and the effect of global stressors on eating disorders among medical students. Front Psychol. 2025;16:1507910. 10.3389/fpsyg.2025.1507910. 39968197 10.3389/fpsyg.2025.1507910 PMC11832490

[5] Giddens E, Noy B, Steward T, Verdejo-García A. The influence of stress on the neural underpinnings of disinhibited eating: a systematic review and future directions for research. Rev Endocr Metab Disord. 2023;24:713–34. 10.1007/s11154-023-09814-4. 37310550 10.1007/s11154-023-09814-4 PMC10404573

[6] Bazo Perez M, Frazier LD. Risk and resilience in eating disorders: differentiating pathways among psychosocial predictors. J Eat Disord. 2024;12:62. 10.1186/s40337-024-01023-x. 38773646 10.1186/s40337-024-01023-x PMC11110273

[7] Di Vincenzo M, Arsenio E, Della Rocca B, Rosa A, Tretola L, Toricco R, et al. Is there a burnout epidemic among medical students? Results from a systematic review. Med (Kaunas). 2024;60. 10.3390/medicina60040575. 10.3390/medicina60040575 PMC11052230 38674221

[8] Bergmann C, Muth T, Loerbroks A. Medical students’ perceptions of stress due to academic studies and its interrelationships with other domains of life: a qualitative study. Med Educ Online. 2019;24:1603526. 10.1080/10872981.2019.1603526. 31007152 10.1080/10872981.2019.1603526 PMC6493308

[9] Motorga R, Ionescu M, Nechita F, Micu D, Băluțoiu I, Dinu MM, et al. Eating disorders in medical students: prevalence, risk factors, comparison with the general population. Front Psychol. 2024;15:1515084. 10.3389/fpsyg.2024.1515084. 39850973 10.3389/fpsyg.2024.1515084 PMC11756529

[10] Fekih-Romdhane F, Daher-Nashif S, Alhuwailah AH, Al Gahtani HMS, Hubail SA, Shuwiekh HAM, et al. The prevalence of feeding and eating disorders symptomology in medical students: an updated systematic review, meta-analysis, and meta-regression. Eat Weight Disord. 2022;27:1991–2010. 10.1007/s40519-021-01351-w. 35067859 10.1007/s40519-021-01351-w PMC8784279

[11] Ngan SW, Chern BCK, Rajarathnam DD, Balan J, Hong TS, Tiang K-P. The relationship between eating disorders and stress among medical undergraduate: a cross-sectional study. Open J Epidemiol. 2017;7:85. 10.4236/ojepi.2017.72008.

[12] Okasha A. Mental health in the middle east: an Egyptian perspective. Clin Psychol Rev. 1999;19. 10.1016/s0272-7358(99)00003-3. :917 – 33. 10.1016/s0272-7358(99)00003-3 10547710

[13] Goldberg D. Manual of the general health questionnaire. NFER-Nelson; 1978.

[14] Haddad C, Khoury C, Salameh P, Sacre H, Hallit R, Kheir N, et al. Validation of the Arabic version of the eating attitude test in lebanon: a population study. Public Health Nutr. 2021;24:4132–43. 10.1017/s1368980020002955. 32895080 10.1017/S1368980020002955 PMC10195249

[15] Aldubayan K, Ghafouri K, Mutwalli H, Kutbi HA, Mumena WA. Validity and consistency of the Arabic version of the eating disorder examination questionnaire (EDE-Q) among Saudi adults. Healthc (Basel). 2023;11:10–52. 10.3390/healthcare11071052. 10.3390/healthcare11071052 PMC10094318 37046979

[16] Cohen S. Perceived stress in a probability sample of the united States. In: Spacapan SaO S, editor. The social psychology of health. Newbury Park, CA: Sage Publications, Inc.; 1988. pp. 31–67.

[17] Chaaya M, Osman H, Naassan G, Mahfoud Z. Validation of the Arabic version of the Cohen perceived stress scale (PSS-10) among pregnant and postpartum women. BMC Psychiatry. 2010;10:111. 10.1186/1471-244X-10-111. 21159169 10.1186/1471-244X-10-111 PMC3016315

[18] Barakat S, McLean SA, Bryant E, Le A, Marks P, Touyz S, et al. Risk factors for eating disorders: findings from a rapid review. J Eat Disord. 2023;11:8. 10.1186/s40337-022-00717-4. 36650572 10.1186/s40337-022-00717-4 PMC9847054

[19] Varela C, Hoyo Á, Tapia-Sanz ME, Jiménez-González AI, Moral BJ, Rodríguez-Fernández P, et al. An update on the underlying risk factors of eating disorders onset during adolescence: a systematic review. Front Psychol. 2023;14:1221679. 10.3389/fpsyg.2023.1221679. 38023032 10.3389/fpsyg.2023.1221679 PMC10663237

[20] Iyer S, Shriraam V. Prevalence of eating disorders and its associated risk factors in students of a medical college hospital in South India. Cureus. 2021;13:e12926. 10.7759/cureus.12926. 33654608 10.7759/cureus.12926 PMC7907547

[21] Bizri M, Geagea L, Kobeissy F, Talih F. Prevalence of eating disorders among medical students in a Lebanese medical school: A Cross-Sectional study. Neuropsychiatr Dis Treat. 2020;16:1879–87. 10.2147/ndt.S266241. 32801721 10.2147/NDT.S266241 PMC7414930

[22] Chan YL, Samy AL, Tong WT, Islam MA, Low WY. Eating disorder among Malaysian university students and its associated factors. Asia Pac J Public Health. 2020;32:334–9. 10.1177/1010539520947879. 32787612 10.1177/1010539520947879

[23] Tavolacci MP, Grigioni S, Richard L, Meyrignac G, Déchelotte P, Ladner J. Eating disorders and associated health risks among university students. J Nutr Educ Behav. 2015;47. 10.1016/j.jneb.2015.06.009. 412 – 20.e1. 10.1016/j.jneb.2015.06.009 26363936

[24] Aamir I. Stress level comparison of medical and nonmedical students: A cross sectional study done at various professional colleges in Karachi, Pakistan. Acta Psychopathologica. 2017;03:1–5. 10.4172/2469-6676.100080.

[25] Husnain MA. Stress level comparison of medical and non-medical students: A cross-sectional study done at various professional colleges in Karachi. Pakistan Acta Psychopathol (Wilmington). 2017;3:8. 10.4172/2469-6676.100080.

[26] Girlani JA, Roswiyani R, Satyadi H, editors. Correlation between Level of Stress and Risk of Eating Disorder Symptoms in Early Adult Individuals. 3rd Tarumanagara International Conference on the Applications of Social Sciences and Humanities (TICASH 2021); 2022: Atlantis Press.

[27] Jahrami H, Saif Z, Trabelsi K, Ghazzawi H, Pandi-Perumal SR, Seeman MV. An umbrella review and a Meta-analysis of Meta-analyses of disordered eating among medical students. Alpha Psychiatry. 2024;25:165–74. 10.5152/alphapsychiatry.2024.241515. 38798808 10.5152/alphapsychiatry.2024.241515 PMC11117415

[28] Swamy PDK. Investigating the correlation between eating disorders and perceived stress among working professionals in coimbatore: an empirical study. Afr J Biomed Res. 2024;27:3379–88. 10.53555/AJBR.v27i3S.2946.

[29] Chaudhari B, Tewari A, Vanka J, Kumar S, Saldanha D. (2017) The relationship of eating disorders risk with body mass index, body image and self-esteem among medical students. Ann Med Health Sci Res 7.

[30] Thangaraju SI, Karpagalakshmi R, Arumuganathan S, Usaid S, Devi SS, Sethumadhavan V. A Cross-Sectional study on prevalence of eating disorder and body image disturbance among female undergraduate medical students. J MENT HEALTH HUM BE. 2020;25:53–6. 10.4103/jmhhb.jmhhb_13_20.

[31] Kabakuş Aykut M, Bilici S. The relationship between the risk of eating disorder and meal patterns in university students. Eat Weight Disord. 2022;27:579–87. 10.1007/s40519-021-01179-4. 33881762 10.1007/s40519-021-01179-4

[32] Ali E, Shehata W. Eating disorder risk among medical students at Tanta University, Egypt. Egypt J Community Med. 2020;38:17–23. 10.21608/ejcm.2020.119410.

[33] Zhang R, Liu F, Wang X, Wang S. Towards active health: A study on the relationship between physical activity and body image among college students. Heliyon. 2024;10:e38465. 10.1016/j.heliyon.2024.e38465. 39391503 10.1016/j.heliyon.2024.e38465 PMC11466608

[34] Eck KM, Quick V, Byrd-Bredbenner C. Body dissatisfaction, eating styles, weight-related behaviors, and health among young women in the united States. Nutrients. 2022;14. 10.3390/nu14183876. 10.3390/nu14183876 PMC9505776 36145252

[35] Solmi M, Veronese N, Sergi G, Luchini C, Favaro A, Santonastaso P, et al. The association between smoking prevalence and eating disorders: a systematic review and meta-analysis. Addiction. 2016;111:1914–22. 10.1111/add.13457. 27206671 10.1111/add.13457

[36] Mason TB, Tackett AP, Smith CE, Leventhal AM. Tobacco product use for weight control as an eating disorder behavior: recommendations for future clinical and public health research. Int J Eat Disord. 2022;55:313–7. 10.1002/eat.23651. 34866222 10.1002/eat.23651 PMC8917997

[37] Anderson JK. The relation between disordered eating, stress, and anxiety in first-year college women. University of Northern Iowa; 2019.

[38] El-Akabawy G, Abukhaled JK, Alabdullah DW, Aleban SA, Almuqhim SA, Assiri RA. Prevalence of eating disorders among Saudi female university students during the COVID-19 outbreak. J Taibah Univ Med Sci. 2022;17:392–400. 10.1016/j.jtumed.2022.02.001. 35185445 10.1016/j.jtumed.2022.02.001 PMC8843321

[39] Klatzkin RR, Gaffney S, Cyrus K, Bigus E, Brownley KA. Stress-induced eating in women with binge-eating disorder and obesity. Biol Psychol. 2018;131:96–106. 10.1016/j.biopsycho.2016.11.002. 27836626 10.1016/j.biopsycho.2016.11.002

